# The G. A. R. Hospital Service

**Published:** 1897-09

**Authors:** 


### THE G. A. R. HOSPITAL SERVICE.

WHILE Buffalo scored many triumphs in the entertainment of its distinguished guests, the Grand Army of the Republic and their friends, last month, not the least notable one was the manner in which it cared for the sick comrades. Dr. Ernest

Wende, health commissioner, was early selected to take charge of this service by the citizens' committee, and to say that he performed the duties of his office in a most admirable manner, and to the satisfaction of all, is only to reaffirm what everybody knows.

It is, however, our purpose at this time to mention some of the details of the excellent system adopted and carried out. In the first place a field hospital was established at the corner of Michigan and Wells streets, which, by reason of its location near the principal railway stations, received for the most part emergency cases. It was capable of accommodating 100 patients and was in charge of Dr. Chauncey P. Smith. During the week fifty-three patients were treated at this hospital, the last one being discharged cured on Saturday, August 28th, after which the hospital was closed.

At Camp Jewett, where the hospital was under the immediate charge of Dr. A. H. Briggs, director of hospitals, the principal service was rendered. As an indication of the labor performed we may mention that about 100 patients received treatment during the day of the parade, August 25th. Most of these were cases of sudden illness due to exhaustion or intestinal disturbances. Nevertheless, every available physician and nurse was kept busy from early morning until late evening. At the Terrace hospital, where homeopathic physicians assumed charge, great activity was displayed and excellent management was apparent.

The work done on the day of the parade is always the greatest test of the medical organisation at these meetings. It is to the lasting credit of the Buffalo staff of physicians that they proved equal to the great demand on their energy and skill. Twelve hospital stations were established along the line of march, where were received and treated more than 100 persons who gave out during the parade. Beside this over 5,000 gallons of ice water were dispensed to the marching host.

The ambulance service, under command of Dr. G. W. York, was most admirably managed and rendered prompt as well as careful aid whenever called upon. The part taken by the nurses must not be overlooked, for it was an important one and well performed. It was stated, on whose authority we know not, that Canadian nurses refused to tender their services to the medical department.

As a final remark, we desire to state that only words of praise are due to every physician, nurse and attendant who contributed skill, time and money to the G. A. R. hospital service during the 31st annual encampment at Buffalo, August 23-28, 1897.

